# Isolated soft tissue tuberculosis: a case report and literature review

**DOI:** 10.3389/fmed.2023.1205446

**Published:** 2023-11-15

**Authors:** Baolin Chen, Yuxiang Bao, Jun Chen, Yunpu Zhang, Qifu Wen, Kai Wang, Xiaoming Cheng, Junyuan Lv

**Affiliations:** ^1^Department of General Surgery, The Affiliated Hospital of Zunyi Medical University, Zunyi, China; ^2^Department of Thyroid and Breast Surgery, The Affiliated Hospital of Zunyi Medical University, Zunyi, China; ^3^Department of General Surgery, People's Hospital of Sandu Shui Nationality Autonomous County, Duyun, China; ^4^Department of Pathology, The Affiliated Hospital of Zunyi Medical University, Zunyi, China

**Keywords:** soft tissue tuberculosis, extrapulmonary, diagnosis, treatment, case report

## Abstract

Soft tissue tuberculosis is a rare extrapulmonary form of tuberculosis with limited experience in diagnosis and treatment. Soft tissue tuberculosis is an extrapulmonary infection with atypical clinical symptoms that can be easily misdiagnosed. In this article, we report a case of a female patient with isolated soft tissue tuberculosis who presented with a progressively enlarging subcutaneous mass as the primary symptom, and was suspected of having a subcutaneous lipoma after ultrasonography. A review of the literature revealed that soft tissue tuberculosis is insidious and mainly occurs in muscles and subcutaneous tissues. It was indicated by histopathology and qPCR testing for *Mycobacterium tuberculosis* complex. There is no standard treatment protocol for soft tissue tuberculosis, and a comprehensive regimen of surgical debridement of the lesion combined with chemotherapy can be used following the guidelines for treating extrapulmonary tuberculosis. Early diagnosis and standardized anti-tuberculosis treatment can significantly improve the prognosis of patients.

## Introduction

Tuberculosis is prevalent in underdeveloped and developing nations, including China. Tuberculosis infection occurs primarily in the lung, called pulmonary tuberculosis, but it can also attack other organs or sites outside the lungs causing extrapulmonary tuberculosis (1, 2). A Chinese tuberculosis study reported that extrapulmonary tuberculosis accounts for approximately 24.6% of tuberculosis cases, including the respiratory system (35.5% of extrapulmonary), musculoskeletal system (15.8%), and peripheral lymphatic system (15.8%) (3). Soft tissue tuberculosis is caused by the direct invasion of *Mycobacterium tuberculosis* or by the spread of tuberculosis lesions from other organs to soft tissues, such as muscles, tendons, and subcutaneous tissues via the bloodstream or lymphatic system. It may occur alone or in association with pulmonary tuberculosis.

Isolated soft tissue tuberculosis is relatively rare, accounting for only 1–2% of all cases of pulmonary and extrapulmonary tuberculosis (4). The clinical presentation of soft tissue tuberculosis is variable and can often be ignored by clinicians (5). Herein, we report a case of soft tissue tuberculosis of the forearm with progressive enlargement of a subcutaneous mass as the first symptom. We also review the relevant literature to discuss its clinical features and prognosis.

## Case presentation

The patient was a 40-year-old female admitted to the hospital with a 2 years history of progressive enlargement in the right forearm. The patient had no cough, sputum, hypothermia, night sweats, dyspnea, chest tightness, or fatigue. Physical examination revealed a 6 cm × 3 cm mass was found on the right forearm with a soft texture, no tenderness, and normal skin temperature, mobility, and wrist joint movement. The patient denied any history of local tissue trauma and had no previous history of tuberculosis, immune disorders, tumors, or other systemic diseases.

**Figure 1:**
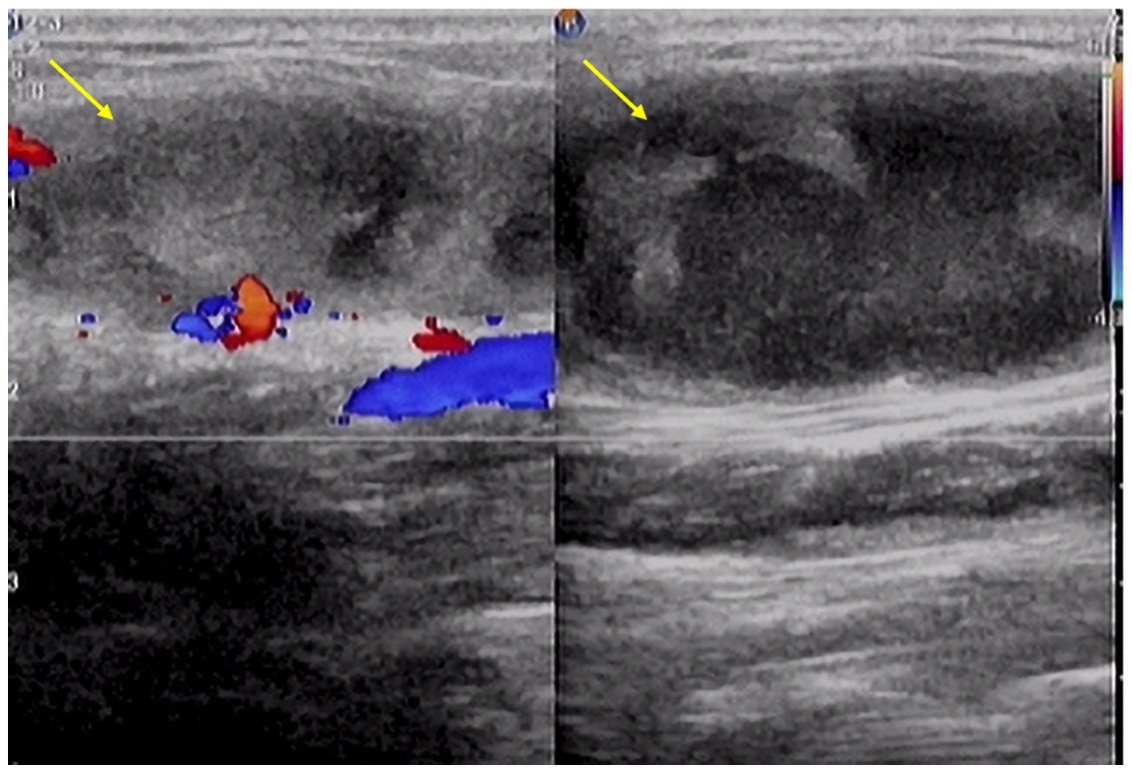
Ultrasonography reveals an irregular mass (arrows) with unclear edge.

**Figure 2:**
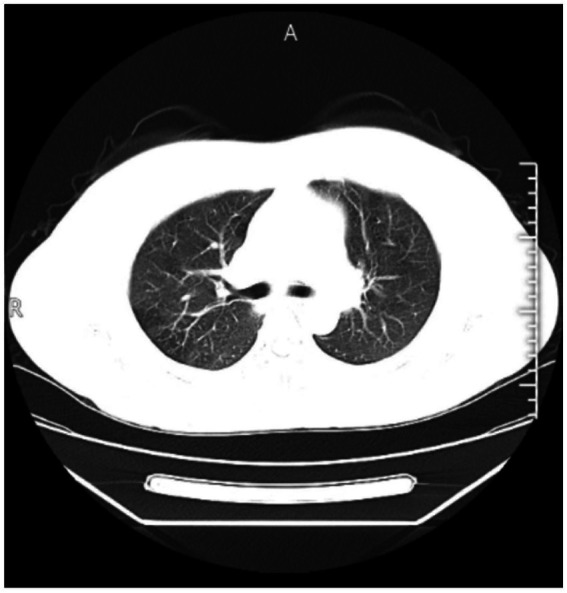
Chest CT scans showed no significant abnormalities.

Superficial ultrasonography of the forearm revealed an irregular mass measuring approximately 56.7 mm × 16.4 mm with unclear borders (Figure 1). CT examination of the chest showed no abnormality (Figure 2). The laboratory blood test revealed a hemoglobin level of 131 g/L, leukocytes of 5.13 × 10^9^/L, neutrophil percentage of 70.6%, lymphocyte percentage of 19.4%, eosinophil percentage of 4.68%, and hematocrit of 20 mm/h. Liver and renal function tests were normal.

**Figure 3:**
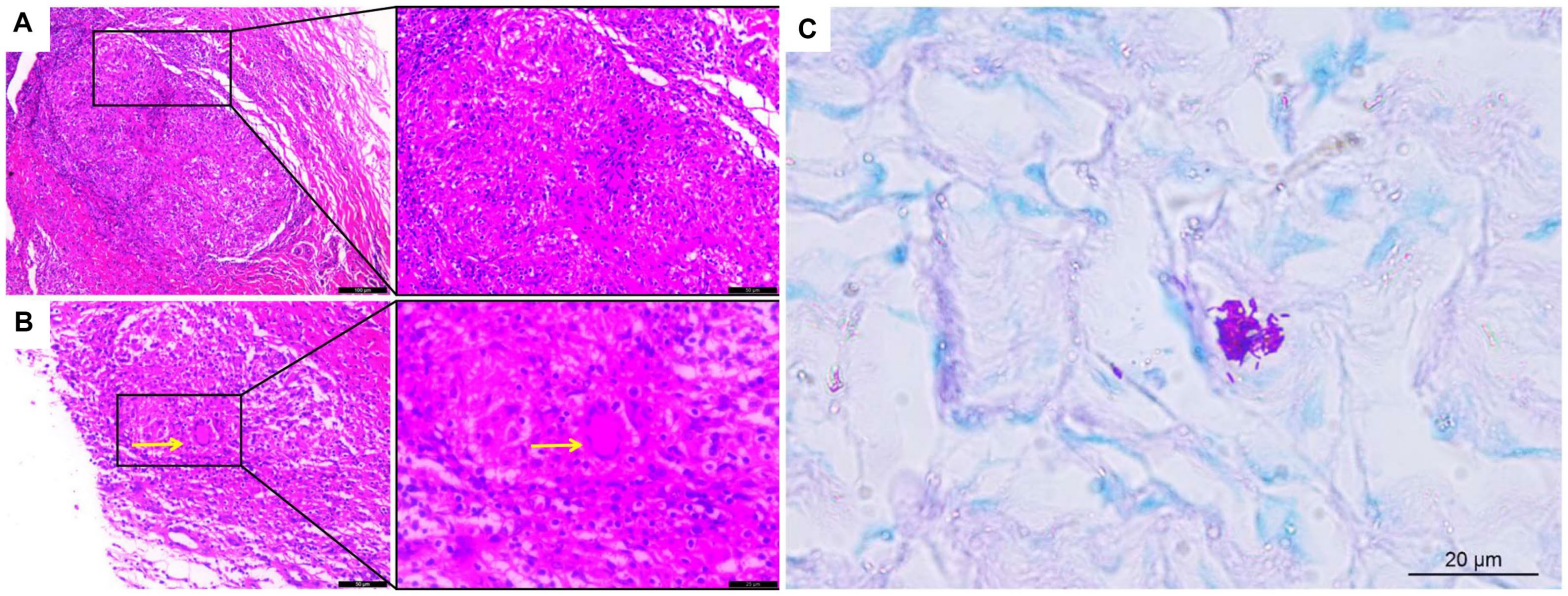
Histological analysis of soft tissue tuberculosis. **(A)** H&E staining showing granulomas with inflammatory cell infiltration and **(B)** multinucleated giant cells (×200); **(C)** Acid-fast bacillus staining was positive. Yellow arrows indicate multinucleated giant cells (×400).

**Figure 4:**
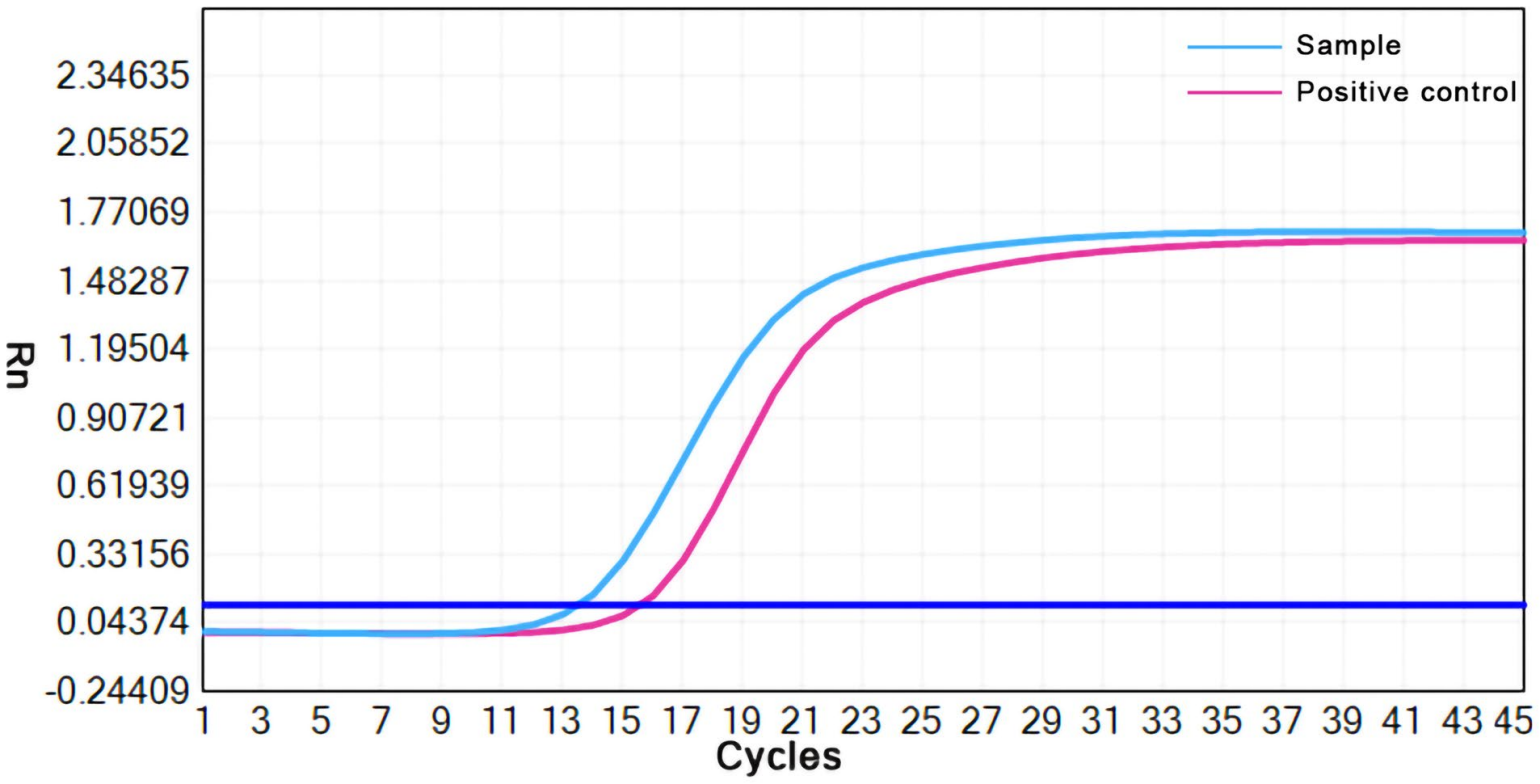
Result of the qPCR assays for the IS6110 gene.

The lipoma diagnosis was established according to the examination results, and the mass was resected on August 12, 2022. The mass was observed intraoperatively as a cystic-solid mass with an incomplete covering, and the mass incision revealed grayish-white fluid with small grayish-white particles like fish eggs. Soft tissue tuberculosis of the right forearm was identified by postoperative pathology, which showed fibrocystic tissue with granulation tissue and granuloma formation, inflammatory cell infiltration (Figure 3A), and multinucleated giant cells in some areas (Figure 3B). Staining for acid fast bacilli were positive (Figure 3C) and the IS6110 gene was detected by quantitative real-time PCR (qPCR) using the *Mycobacterium tuberculosis* Complex kit (Cat.801176, Zeesan Biotech, Xiamen, China), the result showed positive amplification (Figure 4). However, there is no standardized treatment for soft tissue tuberculosis. Considering that the patient lives in a region with a high prevalence of isoniazid resistance (6). The Chinese guideline for diagnosis and treatment of pulmonary tuberculosis suggests that the 2HRZE/4HRE regimen be used in regions where new tuberculosis patients have high prevalence of resistance to isoniazid (7). Finally, we formulated a 2HRZE/4HRE anti-tuberculosis regimen for the patient. It included a 4-drug anti-tuberculosis treatment with isoniazid (H) 300 mg, rifampicin (R) 450 mg, ethambutol (E) 750 mg, and pyrazinamide (Z) 1,500 mg for 2 months, followed by isoniazid (H) 300 mg, rifampicin (R) 450 mg, and ethambutol (E) 750 mg for 4 months. Ten days after the surgery, the patient had good wound healing in the right forearm. After 6 months of anti-tuberculosis treatment follow-up, the patient’s swelling had completely disappeared and the appearance of the right hand was similar to that of the left hand.

## Literature review of soft tissue tuberculosis

### Literature search and screening

We identified an additional 46 cases (40 articles) in the PubMed database from the construction of the database to the present. The medical subject heading (MESH) terms used were as follows: soft tissue tuberculosis, muscular tuberculosis, muscle tuberculosis, musculoskeletal tuberculosis and tubercular pyomyositis. Inclusion criteria: (1) soft tissue tuberculosis. Exclusion criteria: (1) duplicate publications and cohort study literature; (2) non-English language literature; and (3) undetailed case information. The literature was screened independently by 2 investigators according to the exclusion criteria. In case of disagreement, a senior physician in the relevant field was consulted.

### Clinical data of the included studies

A total of 40 eligible studies involving 46 patients with soft tissue tuberculosis were identified. The details of the pertinent cases described in the literature were compiled, including the patients’ general health status (age, sex, location, and medical history), clinical symptoms, site of onset, pathological detection methods, diagnostic techniques, and treatments (Table 1).

**Table 1:** Studies on soft tissue tuberculosis.

Case	Age	Gender	Areas	Underlying disease and pulmonary lesions	Main clinical symptom	Involved sites	Diagnostic approach	Therapy
Franco-Paredes and Blumberg (8)	24	M	Mexico	–	Mass	Left psoas	Bacterial culture	Surgery, 6HREZ + cefazolin 6 weeks
Ergin et al. (9)	49	M	Turkey	CRF	Pain and headache	Left gluteal muscle	Histopathology	Surgery, HZE
Haq et al. (10)	23	M	Angolan	–	Pain and weakness,	Right axilla	Histopathology	Surgery, 6HRZ
Chu et al. (11)	29	F	China	Left otalgia with intermittent otorrhoea	Swelling and tenderness	Left temporal area	FNAB	12HREZ
Tanomkiat and Buranapanitkit (12)	25	M	Thailand	Traumatism	Pain and mass	Right psoas muscle	FNAB	Drainage, 6HR/2ZE
Winzer et al (13)	53	M	Australia	Tuberculosis	Pain and mass	Right pectoral muscle	Histopathology	Surgery
Rajapakse et al. (14)	15	M	Bangladeshi	–	Mass	Left quadratus psoas	FNAB	12HREZ
Trikha et al (15)	30	F	India	–	Swelling and pain	Left vastus lateralis	Histopathology	Surgery, 3HREZ/9RH
Gottschalk et al. (16)	24	F	India	Weight loss	Swelling and pain	Right arm and the base of hemithorax	Histopathology	6HREZ
Khosrovaneh et al. (17)	85	F	USA	Traumas and weight loss	Swelling	Right chest	Histopathology	Surgery, 2HRZ/7HR
Sabat and Kumar (18)	21	F	India	Weight loss and anorexia	Mass	Right rectus femoris	FNAB	2HREZ/4HR_3_
Huang et al. (19)	58	M	China	Primary Sjogren’s syndrome and fever	Swelling and pain	Left thigh	Muscle biopsy	UA
Perez-Alonso et al. (20)	71	M	Spain	Weight loss and anorexia	Swelling and pain	Right thigh	Bacterial culture	Surgery, RE + azithromycin
Arora et al. (21)	15	M	India	Weight loss and anorexia	Swelling	Left thigh	Bacterial culture	2HREZ/4HR_3_
Shields and Robinson (22)	35	F	India	Weight loss and anorexia night sweats	Back pain	Iliopsoas	FNAB	6HREZ
Elshafie et al. (23)	25	M	Oman	–	Swelling	Right gluteal region	FNAB	9HREZ
Lee et al. (24)	62	M	Korea	THA	Mass	Right thigh	Surgical biopsy	Anti-tuberculosis drugs for 6 months
Neogi et al. (25)	11	F	India	–	Swelling	Right quadriceps muscle and left adductor muscles.	Surgical biopsy	Surgery, 2HREZ/4HR_3_
Sökücü et al (26)	37	F	Turkey	–	Swelling and pain	Biceps brachii muscle	Surgical biopsy	Surgery, 2HREZ + other drugs for 7 months
Lai et al. (27)	45	F	China	Weight loss	Swelling and pain	Left rectus femoris muscle	Bacterial culture	Drainage, HREZ
Dhakal et al. (28)	9	F	Nepal	–	Swelling	Forearm, calf and back	Histopathology	Antitubercular
Lombardi et al. (29)	50	F	Brazilian	Weight loss	Swelling and pain	Iliopsoas	Bacterial culture	HREZ
Meena et al. (30)	25	M	India	Fatigue and weight loss	Swelling and pain	Triceps muscle	FNAC+ PCR for Mycobacterium	6HREZ
Grigorakos et al. (31)	38, 24	M	Africa	–	Swelling and pain	The base of hemithorax	FNAB	HREZ
Kotecha et al. (32)	83	M	UK	Paget’s disease	Swelling and pain	Left, thigh	Surgical biopsy	6RH + Rifinah
Sbai et al. (33)	45	M	Tunisia	–	Swelling and pain	Right wrist	Histopathology	Surgery, 2HREZ/6HR
Al-Khazraji et al. (34)	33	F	America	SLE and hormonal therapy	Pain and weakness	Left calf muscle	Histopathology	Surgery, HREZ
Sbai et al. (35)	48	M	Tunisia	Tuberculosis	Swelling	Finger	Histopathology	Surgery, 2HREZ/4HR_3_
	52	F		–				
	49	F		Diabetic				
	63	F		Hypertensive				
	52	F		Diabetic				
	44	F		–				
Alaya and Osman (36)	23	F	Tunisia	–	Swelling and pain	Left thigh	FNAB	Anti-tuberculosis drugs for 12 months
Fataki et al. (37)	35	M	Maroc	–	Pain and fever	Right psoas	Surgical biopsy	Surgery, HREZ + ceftriaxone
Hayoun et al. (38)	29	M	Maroc	–	Swelling	Chest	FNAB	2HREZ/4RH_3_
Manicketh et al. (39)	55	F	India	Tuberculosis	Swelling	Left wrist and right calf	FNAC	UA
Hashimoto et al. (40)	79	M	Japan	–	Swelling	Left wrist and forearm	Incisional biopsy	Surgery, 2HREZ
Moyano-Bueno et al. (41)	29	M	Senegalese	–	Swelling and pain	Right pectoral region	Histopathology	Surgery, HREZ
Zeng et al. (42)	49	M	China	Tuberculosis	Swelling and pain	Thighs and calves	Surgical biopsy	2HREZ/HR
Murugesh et al. (43)	31	M	India	Renal transplant with immuno-suppressants	Fever, pain, and swollen erythematous	Right foot and calf	Surgical biopsy	9HZE
Fahad et al. (44)	45	F	Pakistan	–	Swelling, pain	Right anterior forearm	Histopathology	Surgery, 2HREZ/4HR_3_
Othman et al. (45)	23	M	Saudi Arabia	Tuberculosis glucose-6-phosphate dehydrogenase deficiency	–	Right chest wall	Histopathology	Surgery, 4HREZ
Mohandes et al. (46)	38	F	Syria	–	Back pain and gait difficulty	Psoas	Biopsy	2HREZ/7HR
He et al. (47)	44	M	China	–	Ulceration and swelling	Left thigh	Next-generation sequencing	Surgery, 2HREZ/4HR
Current study	40	F	China	–	Mass	Right forearm	Histopathology	Surgery, 2HREZ/4HRE

R, rifampicin; H, isoniazid; E, ethambutol; Z, pyrazinamide; FNAB, fine-needle aspiration biopsy; FNAC, fine-needle aspiration cytology; M, male; F, female; CRF, chronic renal failure; THA, total hip replacement; SLE, systemic lupus erythematosus; PCR, polymerase chain reaction; UA, unacquirable; −, none.

## Clinical characteristics of patients with soft tissue tuberculosis

### General conditions

The 46 patients included 25 (54.3%) males and 21 (45.7%) females, with ages ranging from 9 to 85 years, including 41 (89.1%) patients aged >20 years. The cases were distributed as follows: 23 cases in Asian countries, 14 cases in African countries, 4 cases in American countries, 4 cases in European countries, and 1 case in Oceanian countries. The medical background ranged from 2 days to 10 years. Five patients had chronic renal failure, primary dry syndrome, Paget’s disease, SLE, and glucose phosphate dehydrogenase syndrome. Four of these patients received long-term steroids treatment. Eight patients had various degrees of weight loss, two had a history of surgery, two had a history of trauma, and five had a history of tuberculosis.

### Site of disease and clinical symptoms

Of the 46 patients, 18 (39.1%) had local swelling or gradually enlarging masses as their primary clinical symptoms (16), 26 (56.5%) experienced various degrees of pain at the site of onset (19), 1 (2.2%) experienced significant motor dysfunction, and 1 (2.2%) had no apparent clinical manifestations. Weight loss, weakness, anorexia, fever, and night sweats were among the symptoms of tuberculosis toxicity in 10 (21.7%) patients (43). Thirty-eight patients (82.6%) had symptoms at a single site, 8 (17.4%) had symptoms at multiple sites, and 28 (60.8%) had lesions in the muscular areas of the extremity joints, including the thighs, calves, forearms, wrists, and feet. Fifteen patients (32.6%) had infections of the gluteus, thoracodorsal and iliopsoas muscles, with one patient having an abscess in the left psoas muscle involving the iliac crest and the left iliac flank, suggesting osteomyelitis. One patient had an interstitial cystic abscess with necrotic debris extending into the breast tissue. Two patients (4.3%) had a mixed axillary soft tissue lesion and one patient (2.2%) had a temporalis lesion. In overview, none of the individuals with tuberculosis infections in the extremities had combined limb dysfunction.

### Diagnosis and treatment

Clinical diagnosis of soft tissue tuberculosis is challenging and mostly depends on pathological biopsy and culture of *Mycobacterium tuberculosis* complex (48). In 45 of the 46 patients tuberculosis was diagnosed by histopathological analysis, the *Mycobacterium tuberculosis* complex culture and qPCR test. One patient received anti-tuberculosis therapy based on clinical features and anti-tuberculosis drug sensitivity data despite being negative on acid-fast bacillus staining and *Mycobacterium tuberculosis* complex culture (29). HE staining was used to stain the infected tissues, which under the microscope revealed typical caseous necrosis, granuloma development, and occasionally Langhans giant cells. Most patients underwent surgery, including puncture drainage, mass excision biopsy, and abscess dissection. The anti-tuberculosis regimen included a 2–4 months treatment with a combination of the first-line anti-tuberculosis drugs isoniazid (H), rifampicin (R), pyrazinamide (Z), and ethambutol (E), followed by consolidation therapy with isoniazid (H) and rifampicin (R) for 4–10 months. Forty-four patients were reported to have received anti-tuberculosis treatment. Three patients (6.5%) had the most prolonged treatment duration of 12 months, and 17 of the 46 patients (36.9%) were on a 2HRZE/4HR regimen. Early standardized anti-tuberculosis therapy significantly improved patients’ prognosis. After receiving anti-tuberculosis therapy, 43 of the 46 patients experienced improvements, two patients died from spontaneous cerebrovascular accidents, severe shock (9), multi-organ failure (19), and the prognosis for one patient was unclear (20).

## Discussion

The 2019 WHO report estimated a total of 10 million new cases of tuberculosis and 1.5 million tuberculosis-related deaths worldwide (49). Soft tissue tuberculosis is a rare form of extrapulmonary infection caused by *Mycobacterium tuberculosis*. It is most common in young and middle-aged people and slightly more frequent in males than females (50). According to the literature review, most patients (95.6%) had localized swelling or masses as their primary symptom, and more than half of patients (60.8%) had localized pain or tenderness. Soft tissue tuberculosis had the highest rate of involvement in the extremities (60.8%). However, fewer people (13.0%) developed symptoms of systemic tuberculosis toxicity.

The etiology of soft tissue tuberculosis remains unclear. Nonetheless, two main mechanisms of infection have been described: endogenous and exogenous. Endogenous tuberculosis results from the spread of infection through the lymphatic and blood circulation from tuberculous lesions in other body organs. Indeed, *Mycobacterium tuberculosis* can easily spread from distant organs via blood or lymph when the body is immunocompromised, such as in HIV infection, diabetes mellitus, renal failure, and long-term use of glucocorticoids (51). On the other hand, exogenous infection is the result of direct invasion of *Mycobacterium tuberculosis*. About a decade ago, there were case reports in China of tuberculosis bacteria multiplying around the injection site after Bacille Calmette-Guerin (BCG) vaccination, resulting in soft tissue tuberculosis (4). However, no comparable cases have been reported in recent years as a consequence of advances in vaccine preparation techniques in China. Similarly, in an early case report, a patient with tuberculosis suffered a traumatic injury to the buttocks, which resulted in the formation of a cold abscess and a diagnosis of tuberculosis. Thus, the hypothesis was proposed that latent *Mycobacterium tuberculosis* in the lung spreads to the gluteal muscles via the bloodstream (52). Obviously, the hypothesis has yet to be proven. Only 2 of the 46 patients reported in the literature had a history of trauma.

Of the 46 case studies analyzed in this study, 29 (63%) were diagnosed by surgical debridement or excisional tissue biopsy, and 17 (37%) were diagnosed by ultrasound or CT-guided puncture biopsy. Microscopic examination of pathological sections from 20 patients (43.5%) showed a significant lymphocytic, neutrophilic, or monocytic infiltration with caseous necrosis or granuloma formation and some multinucleated giant cells. All patients had *Mycobacterium tuberculosis* infection detected by one or more tests, including Gram staining, acid-fast bacilli smear, polymerase chain reaction (PCR), Xpert MTB/RIF test, T-SPOT test, next-generation sequencing (NGS), *Mycobacterium tuberculosis* tissue or puncture fluid culture, and direct *Mycobacterium tuberculosis* test (53). The qPCR or gene detection of *Mycobacterium tuberculosis* is essential for the diagnosis of soft tissue tuberculosis. On the other hand, the acid-fast bacilli smear is less sensitive for detecting *Mycobacterium tuberculosis*, usually 40% sensitive in extrapulmonary tuberculosis (54). This was also demonstrated in our analysis, where the positive rate of the acid-fast bacilli smear was only 33.3%. Due to the insidious nature of soft tissue tuberculosis symptoms and the low positive predictive value of associated tests, a combination of tests is often used for diagnosis in clinical practice (55). The diagnosis of soft tissue tuberculosis in the analyzed patients was based on pathological examination showing inflammatory cell infiltration, granuloma formation, multinucleated giant cells, positive staining for acid fast bacilli, and positive PCR for *Mycobacterium tuberculosis*.

Treatment of extrapulmonary tuberculosis consists mainly of lesion debridement and standardized anti-tuberculous drug therapy. Soft tissue tuberculosis with single-site involvement can be surgically excised. However, in patients with multi-site infections and abscess formation, surgical debridement, puncture, and drainage are often used to reduce the lesions and treat them with a sequential standardized anti-tuberculosis regimen (42). Anti-tuberculosis drug therapy is divided into an intensive and a consolidation phase, with emphasis on early treatment, appropriate dosage, regular and complete course, and combination therapy (56). A combination of at least 2 drugs is used to reduce drug resistance. The latest WHO guidelines recommend a 6 months 2HRZE/4HR regimen as the first-line treatment option for pulmonary tuberculosis without arbitrary extension of intensive therapy. Meanwhile, in areas with a high prevalence of isoniazid resistance, the WHO 2022 guideline recommends a 2HRZE/4HR regimen for newly diagnosed patients with extrapulmonary tuberculosis other than central nervous system, bone or joint tuberculosis (57). However, the 2018 edition of the Chinese guideline and the WHO 2010 guideline recommend a 2HRZE/4HRE regimen (7, 58). The literature review shown that 36.9% of patients on the standard 2HRZE/4HR regimen significantly improved. Considering that the patient lives in a region with a high prevalence of isoniazid resistance and the Chinese guideline, we formulated a 2HRZE/4HRE regimen for the patient. Although 2HRZE/4HRE is recommended for new patients with high levels of isoniazid resistance in both the WHO 2010 guideline and the Chinese guideline, the Chinese guideline does not emphasise that 10.3% of previously treated patients are resistant to ethambutol (compared with 2.5% of new patients), which would reduce the effectiveness of this therapy. This is probably why 2HRZE/4HRE remains the usual regimen in China. Regardless of the chosen optimization strategy, it should be noted that long-term use of anti-tuberculosis drugs can cause serious side effects in the internal organs, such as intestinal necrosis (46), hepatic and renal insufficiency (40). The case report and literature review advances our understanding of soft tissue tuberculosis and treatment methods and perform aggressive anti-tuberculosis treatment against drug-resistant *Mycobacterium tuberculosis*.

## Conclusion

In conclusion, comprehensive treatment with surgical excision combined with anti-tuberculosis therapy should be provided for soft tissue tuberculosis. Tuberculosis drug resistance should be taken into account when developing a regimen. Moreover, the regimen should be based on the results of drug susceptibility testing whenever possible.

## Data availability statement

The original contributions presented in the study are included in the article/supplementary materials, further inquiries can be directed to the corresponding authors.

## Ethics statement

The manuscript is a case report with a literature review. No human studies were carried out in preparing the manuscript. Written informed consent was obtained from the individual(s) for the publication of any potentially identifiable images or data included in this article.

## Author contributions

JL and XC were involved in the conception and design of the work. JL revised the manuscript. JC, YZ, and QW collected the data. KW analyzed the histopathological figures. BC and YB prepared the manuscript. All authors contributed to the article and approved the submitted version.
